# Impact of body roundness index on cognitive decline and cognitive impairment in middle-aged and older adults ≥ 45 years: mediating role of biological aging

**DOI:** 10.1038/s41598-026-54325-2

**Published:** 2026-05-26

**Authors:** Ruixue Song, Fangfei Liu, Wenzhe Yang, Luyang Liu

**Affiliations:** 1 Department of Emergency Medicine, Qilu Hospital of Shandong University, Jinan, Shandong China; 2 Shandong Provincial Clinical Research Center for Emergency and Critical Care Medicine, NMPA Key Laboratory for Clinical Research and Evaluation of Innovative Drug, Medical and Pharmaceutical Basic Research Innovation Center of Emergency and Critical Care Medicine, China’s Ministry of Education, Shandong Provincial Engineering Laboratory for Emergency and Critical Care Medicine, Key Laboratory of Cardiopulmonary-Cerebral Resuscitation Research of Shandong Province, Qilu Hospital of Shandong University, Jinan, Shandong China; 3 Department of Neurology, Qilu Hospital of Shandong University, Jinan, Shandong China; 4 School of Public Health, Tianjin Medical University, Tianjin, China; 5 Medical Department, Shandong Provincial Hospital Affiliated to Shandong First Medical University, Jinan, Shandong China

**Keywords:** Body roundness index, Visceral obesity, Cognitive decline, Cognitive impairment, Biological aging, Diseases, Health care, Medical research, Neurology, Neuroscience, Risk factors

## Abstract

The relationship between Body Roundness Index (BRI) and cognitive function in middle-aged and older adults remains unclear. We aimed to explore the association of BRI with cognitive trajectories and incident cognitive impairment and the mediated effect of biological aging. A subset of 8,595 participants (aged 57.71 ± 8.48 years, 52.71% men) from China Health and Retirement Longitudinal Study (CHARLS) were followed up to 9 years. BRI was calculated based on height and waist circumference. Cognitive function was assessed with neuropsychological tests up to 5 times, and cognitive impairment was defined as scoring 1.5 standard deviations below the mean. Biological aging was evaluated using the Klemera-Doubal method for biological age (KDM-BA). Linear mixed-effects models were used to explore the effect of BRI on the annual change in cognitive function. Cox regression, Laplace regression and restricted cubic spline models were used to assess the association between BRI and the occurrence of cognitive impairment. Mediation analyses was performed to assess the role of biological aging in these relationships. During the follow-up, 1,282 (14.9%) developed cognitive impairment. Higher BRI was also associated with a faster decline of global cognition (*β* = − 0.009, 95% confidence interval [CI]: − 0.017 to − 0.007), especially executive function and temporal orientation over time (all *P* < 0.05). The multi-adjusted hazard ratio (HR) (95% CI) of BRI for cognitive impairment was 1.12 (1.05–1.19). Each unit increased in BRI led to a 0.41-year earlier onset of 15th percentile difference (PD) in cognitive impairment (95% CI: 0.13–0.68). A J-shaped dose–response relationship on the risk of cognitive impairment was identified with an inflection point of BRI at 4.61. The mediation proportion of BRI affecting cognition decline and occurrence of cognitive impairment through KDM-BA after adjustment was 47.18% and 10.19%, respectively. These findings suggest that levated BRI accelerated the progression of cognitive decline and increased the risk of cognitive impairment onset in middle-aged and older adults. Biological age may partially mediate the association.

**Supplementary Information:**

The online version contains supplementary material available at 10.1038/s41598-026-54325-2.

## Introduction

With the rapid aging of the global population, dementia is recognized as one of the most significant global challenges for healthcare and social care systems in the twenty-first century^1^. Cognitive impairment, often serving as a key precursor to dementia onset, represents a transitional stage between normal aging and dementia. In China, the overall prevalence of cognitive impairment in 2018 was 15.5%, representing 38.77 million affected individuals^2^. Given these alarming trends, it is crucial to identify and mitigate risk factors for cognitive impairment at an early stage in order to reduce the occurrence of dementia^3^.

Over the past 30 years, the prevalence of obesity has increased significantly worldwide and has become a major public health concern^4^. Studies have demonstrated a significant association between obesity, as measured by various indices, and cognitive impairment^5,6^. However, with the extensive investigation of body composition, traditional obesity indicators such as body mass index (BMI) have limitations in accurately reflecting fat distribution, because fat distribution and body composition vary dramatically among individuals with the same BMI^7,8^. To better embody fat distribution, the body roundness index (BRI), coined by Thomas et al. as a novel anthropometric measurement, additionally considers waist circumference and can more comprehensively estimate body fat percentage and visceral adipose tissue^9^. BRI was found to be superior to other anthropometric indicators in estimating the risk for various chronic diseases such as cardiometabolic disease, cancer, as well as premature death^10–14^. The relationship between the BRI and cognitive impairment has garnered increasing research interest in recent years; however, the evidence remains inconsistent. Some cross-sectional or retrospective studies have supported a significant negative association between elevated BRI and cognitive function^15^, particularly in older adults and individuals with type 2 diabetes^16,17^, with some indicating a stronger effect in women^18^. In contrast, other studies from rural China and Iran found no significant association^19,20^ or a non-linear, inverted U-shaped relationship^21^. In addition, a US population study reported that BRI was negatively correlated with the cognitive function score but was not significantly associated with cognitive impairment^22^. These discrepancies may be attributed to variations in study populations, cognitive assessment tools, and study designs, which further complicate the interpretation of existing findings. Meanwhile, few studies have explored the association between BRI and cognitive decline in a cohort with long-term follow-up. Thus, further well-designed, prospective studies are needed to clarify the role of BRI in cognitive function.

The pathways linking visceral adiposity to cognitive impairment are complex, with evidence pointing to the roles of systemic inflammation and metabolic dysregulation^23,24^. Compared to chronological age, biological age could provide a more accurate reflection of an individual’s true aging status and susceptibility to age-related diseases^25^. Studies have documented that accelerated biological aging plays a critical role in the progression of cognitive decline and neurodegenerative diseases^26,27^. Furthermore, the alterations and accumulation of metabolites in individuals with obesity may significantly accelerate aging^28^. Despite these insights, the exact role of routine clinical biomarker-based biological aging in the interrelationship between BRI and both cognitive decline and impairment has yet to be fully elucidated.

In the present study, we aimed to systematically examine the longitudinal relationship between BRI and cognitive decline as well as cognitive impairment, and to estimate the mediating role of biological aging in the association of BRI with cognitive decline and impairment using data from a nationally representative cohort study of aging.

## Materials and methods

### Study population

The China Health and Retirement Longitudinal Study (CHARLS) is an ongoing nationally representative longitudinal cohort study of community-living individuals aged 45 years or older in China. The study was approved by the Institutional Review Board of Peking University (Ethics number: IRB00001052-11015), and all participants provided written informed consent^29^. Details of the CHARLS design have been reported previously^29^.

In 2011–2012, a total of 17,708 participants from 10,257 households across 450 communities in 150 counties, living in 28 provinces across China were enrolled and subsequently followed every 2–3 years. In the current study, we used the 9-year survey data for analysis, which included 5 national surveys in 2011 (as baseline), 2013, 2015, 2018, as well as 2020. Of the total population, we excluded 405 participants without basic demographic information (age and sex) or under the age of 45; 3,928 participants with missing data on BRI; 737 participants with cognitive impairment at baseline; 3,057 participants without cognitive function measurements at baseline and 834 participants without cognitive function re-assessments during the follow-up. Of the remaining 8,747 participants, 152 were further excluded due to missing covariate data, leaving 8,595 participants in the final study sample (Supplementary Fig. 1).

### Data collection

All participants underwent structured interviews and physical examinations by trained staff at baseline. Information on demographic characteristics, lifestyle factors, physical examination measurements, clinical conditions and treatment, and cognitive function was collected.

Education was categorized as less than high school, high school or equivalent, as well as college and higher. Weight and height were measured and BMI was calculated as weight in kilograms divided by height in meters squared (kg/m^2^). Smoking status was divided into three groups: never, previous smoker and current smoker. Drinking status was classified into never drinking and drinking. Physical activity was defined as whether participants engaged in vigorous or moderate activities no less than once weekly. In addition, all blood samples such as fasting blood glucose (FBG), random blood glucose (RBG), hemoglobin A1c, creatinine, urea nitrogen, high-sensitivity C-reactive protein, platelets and serum lipids [high-density lipoprotein cholesterol (HDL-C), low-density lipoprotein cholesterol (LDL-C), total cholesterol (TC) and triglycerides (TG)] were taken and measured by trained staff at study entry.

Blood pressure was measured three times in the sitting position. The mean of the 3 values was recorded. Hypertension was defined as systolic blood pressure (SBP) ≥ 140 mmHg or diastolic blood pressure (DBP) ≥ 90 mmHg, or history of hypertension, or use of antihypertensive drugs^30^. Diabetes was defined by any of the following criteria: hemoglobin A1c ≥ 6.5%, FBS ≥ 126 mg/dl, RBG ≥ 200 mg/dl, history of diabetes, or the use of diabetes medication^31^. Dyslipidemia was defined as the presence of at least one or more serum lipid profile abnormalities (TC ≥ 5.2 mmol/L, TG ≥ 1.7 mmol/L, LDL-C ≥ 2.58 mmol/L, HDL-C < 1.03 mmol/L for males and < 1.29 mmol/L for females) according to the Third Report of the National Cholesterol Education Program’s Adult Treatment Panel (NCEP ATP-III) guidelines^32^, history of dyslipidemia, or the use of medication. Chronic lung disease, kidney disease, heart disease and stroke were defined as participants’ self-reports of physician-diagnosed history of disease.

### Body roundness index

Standing height and waist circumference (assessed at the navel using a non-stretchable tape at the end of normal exhalation) of study participants were measured during baseline assessment following standardized protocols. BRI was calculated with the following formula^9^:

$$BRI = 364.2 - 365.5 \times \sqrt {1 - \left( {\frac{{\frac{waist circumference}{{2\pi }}}}{\frac{height}{2}}} \right)^{2} }$$

Higher BRI values indicate greater abdominal fat accumulation. After calculating the BRI values, we divided BRI into quartiles defined as follows: Quartile 1 (Q1, < 3.18), Quartile 2 (Q2, 3.18–3.99), Quartile 3 (Q3, 3.99–4.99) and Quartile 4 (Q4, > 4.99)^33^.

### Cognitive function

Cognitive function was assessed at baseline and each wave, using a composite score based on performance in several cognitive tests adapted from those administered in the United States Health and Retirement Study (HRS), covering episodic memory, executive function and temporal orientation^34^. Episodic memory was evaluated by immediate and delayed recall of 10 words. One point was assigned for each correct word and the maximum score was 20. Executive function was assessed through computational ability and visuospatial ability. Computational ability was assessed by serial 7’s with serially subtracting 7 from 100 five times. The final score was the total number of correct subtractions (range: 0–5). The picture drawing task asked participants to copy a picture of two overlapping pentagons and was scored as correct (1) or incorrect (0) to evaluate visuospatial ability. Temporal orientation was assessed by asking the year, month, date, and the day of the week, with scores ranging from 0 to 4. The global cognition score was the sum of the final scores from each of the cognitive tests, with a higher score indicating better cognitive performance.

All cognitive tests, except for word recall, were consistent in form and administration across the five surveys of data collection. From 2018, the CHARLS investigators harmonized their cognitive measures with HRS by changing the word list and the number of trials for the immediate and delayed word recall task^35^. Thus, we applied a weighted equipercentile equating method to adjust the word recall scores in 2018 and 2020 based on the percentile ranks observed in 2015 to harmonize cognitive scores across surveys^36^. This equating method maps a participant’s relative ranking (percentile) on the newer test versions to the corresponding score at the exact same percentile on the 2015 version. This adjustment ensures that our longitudinal analyses measure true cognitive trajectories rather than artifactual differences caused by changes in test difficulty. Similar approaches for handling raw cognitive scores have been well-embraced in previous studies^36,37^. Cognitive impairment was defined as 1.5 times standard deviation below the mean value^38,39^.

### Biological aging

Biological aging was evaluated using the Klemera-Doubal method-biological age (KDM-BA), which was calculated by combining information from multiple biomarker-specific regressions based on the relationship between biomarker levels and chronological age in a reference population^40^. The KDM-BA algorithm parameters were trained using data from the China Nutrition and Health Survey (CHNS) cohort and then projected onto CHARLS. Considering the availability of the data and correlations with chronological age, a total of eight biomarkers (creatinine, TC, TG, hemoglobin A1c, urea nitrogen, high-sensitivity C-reactive protein, platelets and SBP) were used to estimate KDM-BA for individuals^41^.

### Statistical analysis

Baseline characteristics of the study population by incident cognitive impairment were compared using chi-square tests for categorical variables and *t* tests for continuous variables.

Linear mixed-effects models fitted using Restricted Maximum Likelihood (REML) estimation were used to estimate *β*-coefficients and 95% confidence intervals (CIs) for the associations between BRI and annual change in global cognition and cognitive domains including episodic memory, executive function, and temporal orientation, with follow-up time (in years) as the time scale. The fixed effects included BRI, follow-up time, and their interaction. The random effects included random intercept and slope, allowing the individual differences at baseline and across follow-up. An unstructured covariance matrix was specified for these random effects to allow their variances and covariances to be freely estimated. Considering the possibility of non-linear association between BRI and cognitive trajectories, we additionally tested a quadratic mixed-effects model and a mixed-effects model with splines, which showed no evidence of quadratic curve and splines (data were not shown).

Cox regression models were used to estimate hazard ratios (HRs) and 95% CIs for cognitive impairment associated with BRI (as continuous and categorical). The proportional hazards assumption was tested when the Cox regression was employed and no violation of the assumption was observed. Given the overall cumulative incidence rate was approximately 15%, the Laplace regression models were used to evaluate the 15th percentile differences (PDs) in cognitive impairment onset time in relation to BRI (as continuous and categorical), ensuring robust estimation bounded within the observed empirical event threshold. CIs for the PDs were estimated using asymptotic analytical standard errors. This provides a complementary approach to Cox regression models, as it directly quantifies differences in survival time percentiles, offering clinically interpretable metrics^42^.

Restricted cubic spline (RCS) models were used to explore the dose–response relationship between BRI and the risk of cognitive impairment and identify potential breakpoints. The number of knots varying from 3 to 7 were tested respectively, and the model with the lowest Akaike information criterion (AIC) value was selected. The log-likelihood ratio test (LRT) was also used to determine if the piecewise model provided a significantly better fit than the standard Cox regression model. We also estimated the dose–response relationship between the BRI and the risk of cognitive impairment stratified by age (< 65 vs. ≥ 65).

We conducted a mediation analysis to assess the potential mediating effects of KDM-BA on the association of BRI with global cognitive decline and cognitive impairment in 6,381 participants with data of KDM-BA. The pathways were tested using linear mixed effects model for cognition decline and Cox regression model for cognitive impairment risk. All mediation analyses were executed using the *mediation* and *CMAverse* packages in R. Bootstrapping methods with 1000 iterations were performed to estimate the 95% bias-corrected CIs for the total effect, direct effect, indirect effect, and proportion mediated.

All the models were first adjusted for nothing and then additionally adjusted for potential confounders including age, sex, education, BMI, smoking status, drinking status, physical activity, hypertension, diabetes, dyslipidemia, chronic lung disease, kidney disease, heart disease and stroke.

In supplementary analyses, subgroup Cox regression models of the association between BRI and cognitive function were conducted using stratification factors including age, gender, education, hypertension, diabetes, dyslipidemia, chronic lung disease, kidney disease, heart disease and stroke. Interaction tests were applied to evaluate the consistency of this association across different groups. Considering that death may be a competing risk to the present findings, we estimated the association of BRI-cognitive impairment using Fine and Gray models. Additionally, to address missing values in covariates for 152 participants, Cox regression analyses was conducted using data after multiple imputation by chained equations. In RCS models, subgroup analyses were also performed in participants stratified by gender.

A 2-tailed *P* value of < 0.05 was considered statistically significant for each analysis. All analyses were performed with Stata SE, version 15.0 (Stata Corp LP., College Station, TX) and R software, version 4.2.2 (R Foundation for Statistical Computing, Vienna, Austria).

## Results

### Characteristics of the study population at baseline

The mean age of 8,595 participants (52.71% men) was 57.71 ± 8.48 years. The mean follow-up period was 7.24 years. Participants with incident cognitive impairment were more likely to be older and female to have lower education and lower alcohol consumption to have higher BRI and KDM-BA; as well as a higher prevalence of hypertension at study entry (Table 1). In addition, compared to participants with the lowest BRI quartile (Q1), those with the highest (Q4) were more likely to be older and female; to have lower education and lower levels of physical exercise, less current smoking and drinking, higher KDM-BA; and more likely to have hypertension, diabetes, dyslipidemia, heart disease, stroke and cognitive impairment (Supplementary Table 1). Furthermore, as shown in Supplementary Table 2, compared to the included analytical sample, participants who were excluded due to missing cognitive re-assessments during follow-up were more likely to be older, have hypertension, diabetes, dyslipidemia, chronic lung disease, heart disease, stroke, and exhibit poorer baseline cognitive performance.

**Table 1:** Baseline characteristics of the study population by incident cognitive impairment (n = 8,595).

Characteristic	Cognitive impairment-free n = 7,313	Incident cognitive impairment n = 1,282	*P* value
Age (years), Mean ± SD	57.00 ± 8.21	61.77 ± 8.86	< 0.001
Female, n (%)	3,350 (45.81%)	715 (55.77%)	< 0.001
BMI, Mean ± SD	23.92 ± 4.05	23.04 ± 4.13	< 0.001
Education, n (%)			< 0.001
Less than high school	6,131 (83.84%)	1,257 (98.05%)	
High school or equivalent	995 (13.61%)	23 (1.79%)	
College and higher	187 (2.56%)	2 (0.16%)	
Smoking status, n (%)			0.125
Current smoker	2,467 (33.73%)	397 (30.97%)	
Previous smoker	696 (9.52%)	120 (9.36%)	
Never	4,150 (56.75%)	765 (59.67%)	
Drinking, n (%)	3,117 (42.62%)	505 (39.39%)	0.031
Physical exercise, n (%)	2,156 (29.48%)	372 (29.02%)	0.460
Hypertension, n (%)	2,825 (38.63%)	566 (44.15%)	< 0.001
Diabetes, n (%)	2,729 (37.32%)	478 (37.29%)	0.983
Dyslipidemia, n (%)	5,375 (73.50%)	893 (69.66%)	0.004
Chronic lung disease, n (%)	692 (9.46%)	130 (10.14%)	0.447
Kidney disease, n (%)	472 (6.45%)	75 (5.85%)	0.414
Heart disease, n (%)	842 (11.51%)	124 (9.67%)	0.054
Stroke, n (%)	131 (1.79%)	21 (1.64%)	0.701
BRI, Mean ± SD	4.11 ± 1.51	4.20 ± 1.69	0.044
KDM-BA (years), Mean ± SD*	57.33 ± 9.28	61.77 ± 9.69	< 0.001

Abbreviations: BMI = Body Mass Index; BRI = Body Roundness Index; KDM-BA = The Klemera-Doubal method for biological age; SD = Standard Deviation.

*n = 6,381.

### Relationship between BRI and cognitive decline

**Fig. 1:**
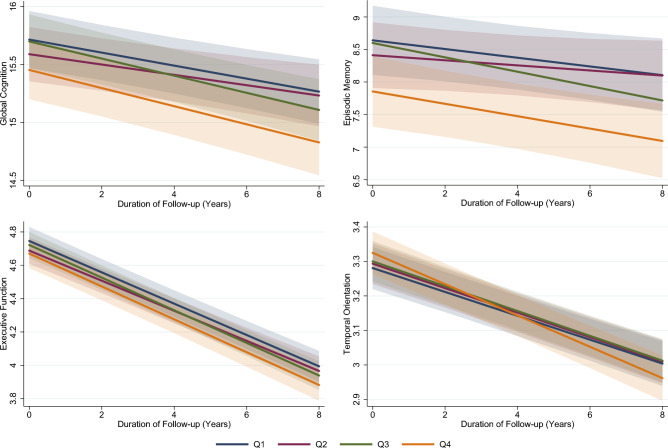
Cognitive trajectories in global cognition and different domains by Body Roundness Index (BRI) in quartiles. *Note:* Trajectories represent *β*-coefficients from linear mixed-effects models adjusted for age, sex, education, BMI, smoking status, drinking status, physical activity, hypertension, diabetes, dyslipidemia, chronic lung disease, kidney disease, heart disease and stroke, with the lowest (Q1) as reference group.

In a multi-adjusted mixed effects model where BRI was treated as a continuous variable, higher BRI was associated with worse baseline global cognition (*β* = − 0.039, 95% CI: − 0.0076 to − 0.001) and episodic memory (*β* = − 0.026, 95% CI: − 0.044 to − 0.008), and a faster annual rate of decline in global cognition (*β* = − 0.009, 95% CI: − 0.017 to − 0.001), executive function (*β* = − 0.004, 95% CI: − 0.008 to − 0.000) and temporal orientation (*β* = − 0.005, 95% CI: − 0.009  to− 0.001) after adjustment. When BRI was used as a categorical variable, the highest BRI was associated with faster global cognitive decline compared with the lowest (*β* = − 0.029, 95% CI: − 0.062 to − 0.004). Moreover, participants with the highest BRI had accelerated decline in executive function (*β* = − 0.005, 95% CI: − 0.022 to − 0.011) and temporal orientation (*β* = − 0.022, 95% CI: − 0.040 to − 0.005) over the follow-up, compared with those with the lowest BRI (Table 2, Fig. 1).

**Table 2:** *β*-coefficients and 95% CIs for the association of the Body Roundness Index (BRI) with the changes of global cognitive function and cognitive function in different domains (n = 8,595).

BRI	Global cognition	Episodic memory	Executive function	Temporal orientation
	*β* (95% CI) *	*β* (95% CI) *	*β* (95% CI) *	*β* (95% CI) *
Baseline				
Continuous BRI	– 0.039 (– 0.076 to – 0.001) ^†^	– 0.026 (– 0.044 to – 0.008) ^†^	– 0.013 (– 0.032 to 0.005)	0.001 (– 0.017 to 0.019)
Categorical				
Q1	Reference	Reference	Reference	Reference
Q2	– 0.044 (– 0.167 to 0.080)	– 0.028 (– 0.090 to 0.033)	– 0.043 (– 0.105 to 0.019)	0.017 (– 0.044 to 0.079)
Q3	0.028 (– 0.104 to 0.160)	0.005 (– 0.060 to 0.070)	– 0.011 (– 0.078 to 0.055)	0.025 (– 0.039 to 0.090)
Q4	– 0.045 (– 0.197 to – 0.107) ^†^	– 0.073 (– 0.145 to – 0.001) ^†^	– 0.044 (– 0.119 to 0.032)	0.068 (– 0.005 to 0.140)
Longitudinal				
Continuous BRI × time	– 0.009 (– 0.017 to – 0.001) ^†^	– 0.002 (– 0.006 to 0.002)	– 0.004 (– 0.008 to – 0.000) ^†^	– 0.005 (– 0.009 to – 0.001) ^†^
Categorical				
Q1 × time	Reference	Reference	Reference	Reference
Q2 × time	0.003 (– 0.029 to 0.036)	0.006 (– 0.012 to 0.023)	0.005 (– 0.011 to 0.022)	– 0.002 (– 0.020 to 0.014)
Q3 × time	– 0.018 (– 0.051 to 0.016)	– 0.009 (– 0.026 to 0.009)	– 0.005 (– 0.021 to 0.012)	– 0.004 (– 0.021 to – 0.013)
Q4 × time	– 0.029 (– 0.062 to – 0.004) ^†^	– 0.006 (– 0.023 to 0.012)	– 0.005 (– 0.022 to – 0.011) ^†^	– 0.022 (– 0.040 to – 0.005) ^†^

* Adjusted for age, sex, education, BMI, smoking status, drinking status, physical activity, hypertension, diabetes, dyslipidemia, chronic lung disease, kidney disease, heart disease and stroke.

^†^ *P* < 0.05.

### Association between BRI and incident cognitive impairment

Over 42,975 person-years of follow-up, 1,282 participants developed cognitive impairment. In both crude and multi-adjusted Cox regression models, a higher BRI (as a continuous variable) was significantly associated with an increased risk of cognitive impairment (multi-adjusted HR = 1.12, 95% CI: 1.05–1.19). Compared to those in the Q1 group, the multi-adjusted HR (95% CIs) of incident cognitive impairment of Q4 was 1.53 (1.22–1.93) (Table 3).

**Table 3:** Hazard ratios (HRs), 95% confidence intervals (CIs), and 15th percentile difference (PDs) in years of incident cognitive impairment in relation to Body Roundness Index (BRI) (n = 8,595).

BRI	No. of subjects	No. of cases	Cox Regression		Laplace Regression	
			HR (95% CI)	HR (95% CI) *	15th PDs (y) (95% CI)	15th PDs (y) (95% CI) *
Continuous	8,595	1,282	1.04 (1.01 to 1.08) ^†^	1.12 (1.05 to 1.19) ^†^	– 0.10 (– 0.19 to – 0.01)^†^	– 0.41 (– 0.68 to – 0.13)^†^
Categorical						
Q1	2,149	330	Reference	Reference	Reference	Reference
Q2	2,149	319	0.95 (0.82 to 1.11)	1.09 (0.93 to 1.28)	0.44 (– 3.79 to 4.67)	– 0.26 (– 1.05 to 0.52)
Q3	2,149	288	0.87 (0.74 to 1.02)	1.12 (0.93 to 1.36)	0.99 (– 1.76 to 3.74)	– 0.42 (– 1.41 to 0.56)
Q4	2,148	345	1.06 (0.91 to 1.23)	1.53 (1.22 to 1.93) ^†^	– 0.99 (– 3.74 to 1.76)	– 1.47 (– 2.66 to – 0.28)^†^

* Adjusted for age, sex, education, BMI, smoking status, drinking status, physical activity, hypertension, diabetes, dyslipidemia, chronic lung disease, kidney disease, heart disease and stroke.

^†^ *P* < 0.05.

Laplace regression analysis showed that the multi-adjusted 15th PD (95% CI) of time (years) at incident cognitive impairment for the participants in Q4 group was 1.47 (0.28–2.66) years earlier than those in Q1 group. Each unit increase in BRI led to a 0.41-year earlier onset of cognitive impairment (Table 3).

**Fig. 2:**
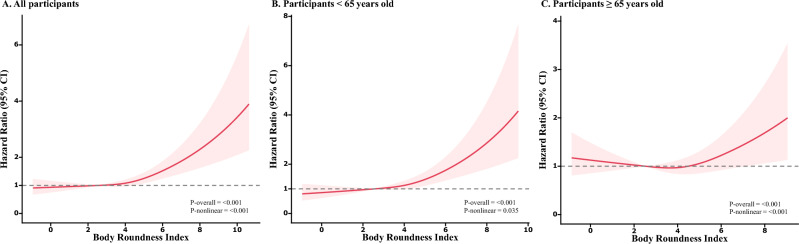
Spline curves for the association between Body Roundness Index (BRI) and cognitive impairment. *Note:* A: Adjusted for age, sex, education, BMI, smoking status, drinking status, physical activity, hypertension, diabetes, dyslipidemia, chronic lung disease, kidney disease, heart disease and stroke. B & C: Adjusted for sex, education, BMI, smoking status, drinking status, physical activity, hypertension, diabetes, dyslipidemia, chronic lung disease, kidney disease, heart disease and stroke. Adjusted HRs of cognitive impairment from RCS Cox regression models with knots at the 5th, 35th, 65th, and 95th percentiles. The solid lines and bands represent the HRs and corresponding 95% CIs.

We used RCS with 4 knots at the 5th, 35th, 65th and 95th percentiles. RCS curve revealed a J-shaped relationship between BRI and cognitive function (*P*-nonlinear < 0.001; Fig. 2A). Further, piecewise regression revealed distinct associations across BRI segments. Specifically, as the values of BRI increased, the curve exhibited a nonsignificant upward trend (HR = 1.05, 95% CI: 0.96–1.14) for BRI values below 4.61. However, the slope of the curve appeared to steepen when BRI exceeded 4.61, suggesting that higher levels of BRI were associated with a progressively greater hazard, with each unit increase in BRI leading to a 24% higher risk of cognitive impairment (HR = 1.24, 95% CI: 1.13–1.35). The LRT indicated that the piecewise model provided a significantly better fit than the conventional model (*P* = 0.011) (Supplementary Table 3). We further performed stratified analysis by age, and found that BRI and cognitive function of participants under 65 years old showed a significant J-shaped relationship (*P*-nonlinear = 0.035) after multi-adjustment (Fig. 2B), and the curve inflection point of multi-factor correction was 5.44. The J-shaped association was also significant in people aged 65 years and older (*P*-nonlinear < 0.001; Fig. 2C), and the curve inflection point was 4.31.

### The mediating role of biological aging

**Fig. 3:**
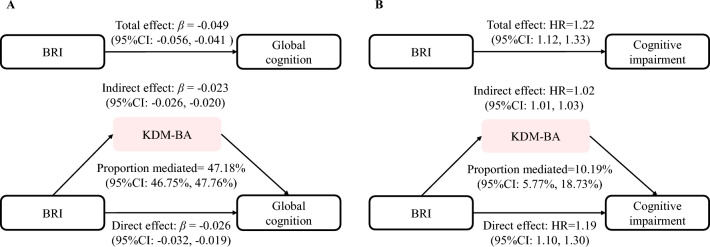
The mediating role of biological age in the association of Body Roundness Index (BRI) with global cognitive decline and cognitive impairment. *Note:* Adjusted for age, sex, education, BMI, smoking status, drinking status, physical activity, hypertension, diabetes, dyslipidemia, chronic lung disease, kidney disease, heart disease and stroke.

The mediating analysis revealed the significant mediating effects of biological aging on the association of BRI with cognitive decline and cognitive impairment (all *P* < 0.001). After adjusting for covariates, the proportion mediated by KDM-BA for the association of BRI with cognitive decline and the occurrence of cognitive impairment was 47.18% and 10.19%, respectively (Fig. 3).

### Supplementary analysis

Subgroup Cox regression analyses showed that the association between BRI and cognitive impairment remained significant in almost all subgroups (Supplementary Fig. 2). Furthermore, interaction effect was not significant, suggesting there was no joint influence of these factors with BRI on the risk of cognitive impairment (data not shown). The association of BRI with cognitive impairment was not substantially altered when adjusting for the competing risk of mortality during the follow-up or including participants with imputed missing values (Supplementary Table 4 and Supplementary Table 5). In RCS analysis stratified by gender, significant J-shaped correlation was found in both male and female participants (Supplementary Fig. 3), the curve inflection points were 4.63 and 5.27, respectively.

## Discussion

In this large-scale, nationally representative longitudinal study of middle-aged and older Chinese adults, we found that elevated baseline BRI was significantly associated with a faster rate of decline in global cognition, executive function and temporal orientation, an increased risk of incident cognitive impairment, and an earlier time to its onset over a 9-year follow-up. A J-shaped dose–response relationship was also identified between BRI and cognitive impairment, with a threshold effect observed at a BRI value of 4.61, beyond which the risk rose markedly. In addition, biological age plays a mediating role in the BRI-cognition association. These findings suggest that BRI, a novel anthropometric index reflecting visceral adiposity, may serve as a valuable and practical indicator for identifying individuals at high risk of cognitive disorder.

Adipose tissue is not only an organ storing energy, but also involved in endocrine functions^43^. Excessive accumulation of adipose tissue may cause a pro-inflammatory state and endocrine dysfunction then consequently leading to a number of serious health consequences^44^. Abdominal obesity is correlated with extensive visceral adipose tissue and characterized by the accumulation of fat around the abdomen, which poses a more pronounced health threat than general obesity^45^. It is also a prominent feature of obesity in Asian populations^46,47^. Compared with traditional obesity indices like BMI, the superiority of BRI lies in its ability to better capture body fat distribution, particularly visceral adipose tissue, which is a known contributor to metabolic dysregulation^48^. Changes in body composition such as the loss of muscle mass and bone mineral density in aging populations may render BMI less informative^49–51^, while BRI incorporates waist circumference and height, providing a more accurate estimate of overall body fat percentage and central obesity^9^.

Several previous studies have reported negative associations between BRI and cognitive function and our results are consistent with these findings. For instance, two studies from the United States and China both found that higher BRI levels were correlated with poorer cognitive performance in older adults and individuals with type 2 diabetes, respectively^16,17^. Similarly, Liu et al. reported a positive association between BRI and cognitive impairment among women with type 2 diabetes^18^. The consistency across these studies underscores the potential role of visceral obesity in cognitive aging. However, it is important to acknowledge the discrepancies with other research. For example, Ramezani Kashal et al. found no significant association between BRI and cognitive impairment in an Iranian elderly cohort^20^, similar to the findings reported in older adults of rural China and the United States^19,22^, while one study described an inverted U-shaped relationship in a rural Chinese population^21^. These inconsistencies may be attributed to differences in study populations (e.g., age, health status, country), cognitive assessment tools (such as Mini-Mental State Examination [MMSE], Six Item Cognitive Impairment Test [6-CIT], or Digit Symbol Substitution Test [DSST]), or study designs (cross-sectional vs. longitudinal).

In the present study, we found that elevated BRI was associated with an increased risk and an earlier onset of incident cognitive impairment. Inconsistent with previous research, the association was not linear but followed a threshold-based pattern. This nonlinear dose-response pattern suggests the existence of a critical threshold beyond which visceral adiposity exerts markedly stronger detrimental effects on cognitive health. Furthermore, we also found that higher BRI accelerated the progression of cognitive decline, especially in executive function and temporal orientation, which had not been reported in relevant research. It is worth acknowledging that although the longitudinal effect sizes computed for continuous cognitive variables (i.e., annual rate of decline) seem numerically modest, their clinical implications are substantial. In the context of population health, even a small increase in the annual rate of decline over a 9-year period can accumulate into a meaningful loss of cognitive function. This accelerated, subclinical deterioration can eventually push aging individuals across the threshold into mild cognitive impairment or dementia years earlier than they otherwise would have experienced. Our study adds to these discoveries by employing a prospective design with repeated cognitive measures, multiple analytical methods, and a large sample that enhances the reliability and generalizability of the findings. While recent evidence has utilized conceptually parallel indices like the Visceral Adiposity Index (VAI) to predict cognitive outcomes^39,52^, VAI inherently relies on fasting clinical laboratory tests (e.g., triglycerides and HDL). In contrast, our findings strongly support the utility of BRI as a fully non-invasive, zero-cost anthropometric screening tool. Demonstrating that an easily accessible metric, which can be utilized for home-based self-monitoring and telemedicine, robustly predicts the long-term risk of cognitive decline highlights its distinct practical utility. It shifts the paradigm of cognitive risk screening from clinical blood tests to continuous, everyday preventative management. Specifically, guided by our non-linear threshold analysis, individuals with a BRI above 4.61 may benefit from more vigilant monitoring and lifestyle interventions aimed at reducing visceral fat (e.g., physical activity, dietary modifications), alongside the management of associated metabolic conditions.

The biological mechanisms underlying the association of high BRI with cognitive impairment and cognitive decline are multifactorial. Mediation analysis identified biological aging as a significant mediator linking visceral fat accumulation to cognitive decline and the occurrence of cognitive impairment. This provides a mechanistic explanation for the link between the visceral fat accumulation and brain health.

Visceral adipose tissue is metabolically active and secretes various adipokines and pro-inflammatory cytokines such as tumor necrosis factor-alpha (TNF-α) and interleukin-6 (IL-6)^53^, thereby inducing systemic chronic inflammation that accelerates aging and increases an individual’s susceptibility to various adverse consequences. Aligning closely with our hypothesis, a study with a large and diverse sample of older adults in the United States based on HRS has elegantly demonstrated that systemic inflammation actively accelerates biological clocks, wherein the biological aging acts as a crucial mediator translating inflammatory burden into accelerated cognitive decline and incident dementia^54^. This indicates that systemic inflammation are not only directly damages neurological health but also mediated by accelerating biological aging, which is consistent with our findings on BRI and cognitive decline. Meanwhile, research has shown that KDM-BA is associated with increased activation of pro-inflammatory pathways^55^. Furthermore, KDM-BA could reflect the clinical characteristics of multiple organs and systems resulting from obesity-related metabolic disorders^28,56^. Crucially, our exploratory analyses revealed that no single biological marker, such as hs-CRP or specific metabolic indices alone, could independently mediate this relationship. This critical finding underscores that KDM-BA captures a systemic, synergistic accumulation of subclinical dysregulation across multiple organs, rather than reflecting a single isolated pathway.

The cumulative burden of this multisystem dysregulation and chronic inflammation may contribute to cerebrovascular damage, reduced brain perfusion, white matter lesions, and ultimately, neuronal dysfunction and cognitive decline^23^. These mechanisms promote each other and form a vicious cycle, jointly accelerating the neurodegenerative process. These findings further elucidate the pathways through which visceral fat accumulation impacts cognitive function, and provide statistical epidemiological evidence for subsequent mechanistic studies. Ultimately, integrating the morphological risk threshold with the systemic aging mechanism provides a unified clinical insight: timely lifestyle interventions targeting abdominal obesity may preserve late-life cognition fundamentally by decelerating the biological aging clock.

There are several strengths to this study. First, this was a longitudinal study with a nationally representative sample of middle-aged and older adults in China. The sample includes Chinese adults living in rural and urban areas, which expands the representation of the evidence base on visceral obesity and cognitive aging to these rapidly aging yet underrepresented Asian population groups. Second, we had longitudinal data over a 9-year follow-up period to help establish the temporality and validity of the associations under study. Furthermore, the data collected are of high quality. Anthropometric data and questionnaire information were collected by trained medical professionals. The measurement of biochemical indexes followed standard protocols. Notably, our study utilized in-depth neuropsychological assessments with repeated measures that were harmonized with those used in the HRS and several of its international partner studies around the world. Regarding the exposure metrics, our study highlighted the distinct utility of BRI in older adults. As elderly individuals undergo age-related height loss, waist circumference alone becomes a biased metric, whereas BRI’s geometric model dynamically corrects for these somatic changes. Our head-to-head comparison demonstrated that BRI yielded a statistically superior predictive value for cognitive impairment (area under the curve [AUC] = 0.737) compared to waist circumference alone (AUC = 0.712) and waist-to-height ratio (AUC = 0.713).

However, there are several limitations. First, these results can only be generalized to the Asian populations, as the ethnic, biological and environmental differences exist between Asian and Western populations. Additionally, although we adjusted for an extensive array of socio-demographic and clinical covariates, residual confounding due to unmeasured factors (such as unmeasured genetic or early-life environmental factors) cannot be completely ruled out. Therefore, the mediation results should be interpreted as strong associations indicative of potential mechanisms rather than definitive causal proofs. Third, requiring valid cognitive function re-assessments during follow-up may introduce survivorship or attrition bias. Participants suffering from poor health or severe cognitive impairment were more prone to drop out or pass away during the follow-up period, potentially leaving a biased “healthier” survivor sample. Consequently, our findings might inherently underestimate the true detrimental impact of visceral adiposity on cognition. Finally, although BRI mathematically optimizes body shape estimation, it remains an anthropometric proxy and cannot perfectly distinguish visceral from subcutaneous fat like magnetic resonance imaging.

## Conclusions

Elevated BRI accelerated the progression of cognitive decline, increased the risk of cognitive impairment and advanced its onset over time in middle-aged and older adults. Biological age may partially mediate this association. Our findings suggest that BRI could be a useful screening tool to identify middle-aged and older adults at elevated risk for cognitive impairment from clinical and public health perspective. Future research should focus on investigating the mechanisms through which visceral fat affects brain health, and examining whether interventions targeting BRI reduction can effectively mitigate the risk of incident cognitive impairment and slow down the rate of cognitive decline.

## Supplementary Information

Supplementary Information.

## Data Availability

The original data of this study are available and can be accessed at the following official website: https://charls.pku.edu.cn/en/.

## Abbreviations

AIC: Akaike information criterion
AUC: Area under the curve
BMI: Body mass index
BRI: Body roundness index
CHARLS: China Health and Retirement Longitudinal Study
CI: Confidence interval
DBP: Diastolic blood pressure
FBG: Fasting blood glucose
HDL-C: High density lipoprotein cholesterol
HR: Hazard ratio
HRS: Health and Retirement Study
KDM-BA: The Klemera-Doubal method for biological age
LDL-C: Low density lipoprotein cholesterol
NCEP ATP III: The Third Report of the National Cholesterol Education Program’s Adult Treatment Panel
PD: Percentile difference
REML: Restricted Maximum Likelihood
RBG: Random blood glucose
RCS: Restricted cubic spline
SBP: Systolic blood pressure
TC: Total cholesterol
TG: Triglycerides
