# Supplementary material for: Impact of body roundness index on cognitive decline and cognitive impairment in middle-aged and older adults ≥ 45 years: mediating role of biological aging
Source: Sci Rep. 2026 May 26;16:24031. doi: 10.1038/s41598-026-54325-2 (PMC13439067; doi:10.1038/s41598-026-54325-2)
Supplement: Supplementary file 1 [file 41598_2026_54325_MOESM1_ESM.docx]

**Supplementary Table 1** Baseline characteristics of the study population by Body Roundness Index (BRI) quartile (n = 8,595).

| **Characteristic** | **Q1**  **n = 2,149** | **Q2**  **n = 2,149** | **Q3**  **n = 2,149** | **Q4**  **n = 2,148** | ***P* value** |
| --- | --- | --- | --- | --- | --- |
| Age (years), Mean ± SD | 57.58 ± 8.71 | 57.37 ± 8.30 | 57.42 ± 8.53 | 58.48 ± 8.33 | <0.001 |
| Female, n (%) | 634 (29.50%) | 858 (39.93%) | 1093 (50.86%) | 1480 (68.90%) | <0.001 |
| BMI, Mean ± SD | 20.49 ± 2.76 | 22.42 ± 2.58 | 24.52 ± 2.54 | 27.73 ± 4.10 | <0.001 |
| Education, n (%) |  |  |  |  | 0.002 |
| Less than high school | 1,841 (85.67%) | 1,827 (85.02%) | 1,816 (84.50%) | 1,904 (88.64%) |  |
| High school or equivalent | 267 (12.42%) | 264 (12.28%) | 279 (12.98%) | 208 (9.68%) |  |
| College and higher | 41 (1.91%) | 58 (2.70%) | 54 (2.51%) | 36 (1.68%) |  |
| Smoking status, n (%) |  |  |  |  | <0.001 |
| Current smoker | 1,076 (50.07%) | 803 (37.37%) | 597 (27.78%) | 388 (18.06%) |  |
| Previous smoker | 187 (8.70%) | 217 (10.10%) | 228 (10.61%) | 184 (8.57%) |  |
| Never | 886 (41.23%) | 1,129 (52.54%) | 1,324 (61.61%) | 1,576 (73.37%) |  |
| Drinking, n (%) | 1,099 (51.14%) | 1,009 (46.95%) | 881 (41.00%) | 633 (29.47%) | <0.001 |
| Physical exercise, n (%) | 672 (31.27%) | 666 (30.99%) | 637 (29.64%) | 553 (25.74%) | <0.001 |
| Hypertension, n (%) | 539 (25.08%) | 675 (31.41%) | 938 (43.65%) | 1,239 (57.68%) | <0.001 |
| Diabetes, n (%) | 743 (34.57%) | 753 (35.04%) | 829 (38.58%) | 882 (41.06%) | <0.001 |
| Dyslipidemia, n (%) | 1,273 (59.24%) | 1,460 (67.94%) | 1,691 (78.69%) | 1,844 (85.85%) | <0.001 |
| Chronic lung disease, n (%) | 233 (10.84%) | 218 (10.14%) | 182 (8.47%) | 189 (8.80%) | 0.025 |
| Kidney disease, n (%) | 152 (7.07%) | 135 (6.28%) | 128 (5.96%) | 132 (6.15%) | 0.456 |
| Heart disease, n (%) | 173 (8.05%) | 199 (9.26%) | 253 (11.77%) | 341 (15.88%) | <0.001 |
| Stroke, n (%) | 28 (1.30%) | 25 (1.16%) | 43 (2.00%) | 56 (2.61%) | 0.001 |
| Cognitive score, mean (SD) | 15.69 ± 3.96 | 15.71 ± 4.04 | 15.86 ± 4.06 | 15.54 ± 4.06 | 0.080 |
| Incident cognitive impairment, n (%) | 330 (15.36%) | 319 (14.84%) | 288 (13.40%) | 345 (16.06%) | 0.092 |
| KDM-BA (years), Mean ± SD* | 58.32 ± 9.39 | 57.64 ± 9.40 | 57.70 ± 9.85 | 58.35 ± 9.24 | 0.048 |

Abbreviations: BMI= Body Mass Index; KDM-BA= The Klemera-Doubal method for biological age; SD= Standard Deviation

*n= 6,381

**Supplementary Table 2** Baseline characteristics of the study population with and without cognitive re-assessments during follow-up (n = 9,407).

| **Characteristic** | **Without cognitive function re-assessments**  **n = 812** | **With cognitive function re-assessments**  **n = 8,595** | ***P* value** |
| --- | --- | --- | --- |
| Age (years), Mean ± SD | 62.63 ± 10.80 | 57.71 ± 8.86 | <0.001 |
| Female, n (%) | 394 (48.52%) | 4,065 (47.29%) | 0.503 |
| BMI, Mean ± SD | 23.37 ± 4.03 | 23.79 ± 4.07 | 0.054 |
| Education, n (%) |  |  | 0.002 |
| Less than high school | 714 (87.93%) | 7,388 (85.96%) |  |
| High school or equivalent | 70 (8.62%) | 1,018 (11.84%) |  |
| College and higher | 28 (3.45%) | 189 (2.20%) |  |
| Smoking status, n (%) |  |  | 0.016 |
| Current smoker | 279 (34.36%) | 2,864 (33.32%) |  |
| Previous smoker | 100 (12.32%) | 816 (9.49%) |  |
| Never | 433 (53.33%) | 4,915 (57.18%) |  |
| Drinking, n (%) | 328 (40.39%) | 3,622 (42.14%) | 0.335 |
| Physical exercise, n (%) | 190 (23.40%) | 2,528 (29.41%) | <0.001 |
| Hypertension, n (%) | 400 (49.26%) | 3,391 (39.45%) | <0.001 |
| Diabetes, n (%) | 451 (55.54%) | 3,207 (37.31%) | <0.001 |
| Dyslipidemia, n (%) | 638 (78.57%) | 6268 (72.93%) | 0.001 |
| Chronic lung disease, n (%) | 119 (14.66%) | 822 (9.56%) | <0.001 |
| Kidney disease, n (%) | 63 (7.76%) | 547 (6.36%) | 0.123 |
| Heart disease, n (%) | 126 (15.52%) | 968 (11.26%) | <0.001 |
| Stroke, n (%) | 24 (2.96%) | 152 (1.77%) | 0.017 |
| BRI, Mean ± SD | 4.22 ± 1.55 | 4.12 ± 1.54 | 0.088 |
| KDM-BA (years), Mean ± SD* | 63.91 ± 15.10 | 56.93 ± 11.69 | <0.001 |

Abbreviations: BMI= Body Mass Index; BRI= Body Roundness Index; KDM-BA= The Klemera-Doubal method for biological age; SD= Standard Deviation

*n= 6,830

**Supplementary Table 3** Hazard ratios (HRs) and 95% confidence intervals (CIs) for the association of Body Roundness Index (BRI) with cognitive impairment using two-piecewise Cox regression models (n = 8,595).

| **BRI** | | **HR**  **(95% CI)** | ***P* value** | **HR**  **(95% CI)** ^*^ | ***P* value** |
| --- | --- | --- | --- | --- | --- |
| **< 4.61** | 0.97 (0.92, 1.03) | | 0.368 | 1.05 (0.96, 1.14) | 0.274 |
| **≥ 4.61** | 1.10 (1.05, 1.15) | | <0.001 | 1.24 (1.13, 1.35) | <0.001 |
| **Log likelihood ratio** |  | | 0.026 |  | 0.011 |

^*^ Adjusted for age, sex, education, BMI, smoking status, drinking status, physical activity, hypertension, diabetes, dyslipidemia, chronic lung disease, kidney disease, heart disease and stroke.

**Supplementary Table 4.** Hazard ratios (HRs) and 95% confidence intervals (CIs) for the association of Body Roundness Index (BRI) with cognitive impairment using Fine and Gray models (n = 8,595).

| **BRI** | **No. of subjects** | **Cognitive impairment** | | |
| --- | --- | --- | --- | --- |
|  |  | **No. of cases** | **HR (95% CI)** | **HR (95% CI)** ^*^ |
| Continuous | 8,595 | 1,282 | 1.06 (1.03~1.10) ^†^ | 1.14 (1.06~1.22) ^†^ |
| Categorical |  |  |  |  |
| Q1 | 2,149 | 330 | Reference | Reference |
| Q2 | 2,149 | 319 | 1.04 (0.88~1.22) | 1.13 (0.95~1.35) |
| Q3 | 2,149 | 288 | 0.97 (0.82~1.14) | 1.13 (0.92~1.40) |
| Q4 | 2,148 | 345 | 1.21 (1.03~1.41) ^†^ | 1.49 (1.13~1.96) ^†^ |

^*^ Adjusted for age, sex, education, BMI, smoking status, drinking status, physical activity, hypertension, diabetes, dyslipidemia, chronic lung disease, kidney disease, heart disease and stroke.

^†^ *P*<0.05.

**Supplementary Table 5.** Hazard ratios (HRs) and 95% confidence intervals (CIs) for the association of Body Roundness Index (BRI) with cognitive impairment after multiple imputation by chained equation for 152 participants with missing values on covariates (n = 8,747).

| **BRI** | **No. of subjects** | **Cognitive impairment** | | |
| --- | --- | --- | --- | --- |
|  |  | **No. of cases** | **HR (95% CI)** | **HR (95% CI)** ^*^ |
| Continuous | 8,747 | 1,301 | 1.04 (1.01~1.07) ^†^ | 1.11 (1.05~1.18) ^†^ |
| Categorical |  |  |  |  |
| Q1 | 2,187 | 338 | Reference | Reference |
| Q2 | 2,187 | 321 | 0.94 (0.80~1.09) | 1.07 (0.91~1.26) |
| Q3 | 2,187 | 293 | 0.86 (0.74~1.01) | 1.11 (0.92~1.34) |
| Q4 | 2,186 | 349 | 1.05 (0.90~1.22) | 1.50 (1.19~1.89) ^†^ |

^*^ Adjusted for age, sex, education, BMI, smoking status, drinking status, physical activity, hypertension, diabetes, dyslipidemia, chronic lung disease, kidney disease, heart disease and stroke.

^†^ *P*<0.05.

**Supplementary Figure 1** Flowchart of the study population.

Abbreviations: BRI= Body Roundness Index

**Supplementary Figure 2** Subgroup analyses of incident cognitive impairment in relation to Body Roundness Index (BRI).

Abbreviations: CIs= Confidence Intervals; HRs= Hazard Ratios

**Supplementary Figure 3** Spline curves for the association between Body Roundness Index (BRI) and cognitive impairment.

*Note:* Adjusted for age, education, BMI, smoking status, drinking status, physical activity, hypertension, diabetes, dyslipidemia, chronic lung disease, kidney disease, heart disease and stroke. Adjusted HRs of cognitive impairment from RCS Cox regression models with knots at the 5th, 35th, 65th, and 95th percentiles. The solid lines and bands represent the HRs and corresponding 95% CIs.
